# Audiovisual distraction reduces pain perception during aural microsuction

**DOI:** 10.1308/003588413X13511609955535

**Published:** 2013-01

**Authors:** N Choudhury, I Amer, M Daniels, MJ Wareing

**Affiliations:** Barts Health NHS Trust,UK

**Keywords:** Aural microsuction, Pain, Audiovisual distraction

## Abstract

**Introduction:**

Aural microsuction is a common ear, nose and throat procedure used in the outpatient setting. Some patients, however, find it difficult to tolerate owing to discomfort, pain or noise. This study evaluated the effect of audiovisual distraction on patients’ pain perception and overall satisfaction.

**Methods:**

A prospective study was conducted for patients attending our aural care clinic requiring aural toileting of bilateral mastoid cavities over a three-month period. All microsuction was performed by a single clinical nurse specialist. Any patients with active infection were excluded. For each patient, during microsuction of one ear, they watched the procedure on a television screen while for the other ear they did not view the procedure. All patients received the same real time explanations during microsuction of both ears. After the procedure, each patient completed a visual analogue scale (VAS) to rate the pain they experienced for each ear, with and without access to the television screen. They also documented their preference and reasons why.

**Results:**

A total of 37 patients were included in the study. The mean pain score for patients viewing the procedure was 2.43 compared with a mean of 3.48 for patients with no television view. This difference in patients’ pain perception was statistically lower in the group who observed the procedure on the television (*p*=0.003), consistent with the majority of patients reporting a preference to viewing their procedure (65%).

**Conclusions:**

Audiovisual distraction significantly lowered patients’ VAS pain scores during aural microsuction. This simple intervention can therefore reduce patients’ perceived pain and help improve acceptance of this procedure.

Aural microsuction is one of the most common procedures performed in daily ear, nose and throat (ENT) practice in the outpatient setting. It is used regularly for dewaxing in patients who may have narrow ear canals or large, open mastoid cavities as well as for clearing debris or mucous in infective cases. However, for a considerable number of patients, it can provoke significant anxiety and distress, owing to discomfort and pain as well as the significant noise that it produces.

A variety of different interventions and techniques have been described for some other minimally invasive procedures, with the aim of trying to distract patients and thereby reduce their pain and distress, with variable results. For example, music has been shown to be effective in serving as a useful distraction strategy during colposcopy, significantly reducing patients’ anxiety and pain.^1^ More recently, music has also been found to have a role in reducing intraoperative anxiety levels for patients undergoing minor plastic surgery procedures under local anaesthesia.^2^ However, the effect of visual distraction using three-dimensional video glasses on perceived pain during restorative dental treatment was not found to be significantly different, even though the majority of patients documented a preference for this type of visual distraction technique for future dental treatments.^3^

Audiovisual (AV) distraction, where patients are allowed to view their procedures on a television screen, has also been studied as a distraction technique. AV distraction during colonoscopy has been shown to significantly reduce patients’ pain scores.^4^ Similarly, patients who received AV distraction by viewing their lithotripsy procedure also had significantly lower reported pain scores,^5^ suggesting that it may be a useful adjunct to improve patients’ acceptance for these types of procedures. However, this form of AV distraction for patients undergoing a cystoscopy has also been studied, with variable effects on patients’ pain perception being demonstrated. One study showed a considerable reduction in the pain experienced^6^ but others have shown no significant difference, both in men^7^ and in women.^8^

We sought to evaluate the effect of AV distraction on pain perception for patients undergoing aural microsuction. To our knowledge, this is the first study of its kind to evaluate this for an ENT-based procedure.

## Methods

A prospective, controlled study was conducted to evaluate the effect of AV distraction on patients’ pain scores after undergoing aural microsuction. Patients attending our nurse-led aural care clinic between November 2011 and February 2012 were considered for the study. Only patients with bilateral mastoid cavities, requiring aural microsuction of both cavities, were included, with each patient thereby serving as their own internal control. Any patients with signs of active inflammation or infection in either cavity were excluded so as to avoid the potential for this affecting their pain scores.

All microsuction was performed by a single clinical nurse specialist. Each patient was able to view the microsuction of the left ear on a television screen but was not allowed to view the procedure while microsuction of the right ear was being performed. All patients received the same real time explanations during the procedure for both ears. After the microsuction, patients were asked to complete a simple questionnaire including a ten-point visual analogue scale (VAS) to rate the level of pain experienced during microsuction of each ear in turn. Patients were also asked to document whether they preferred to have microsuction performed with or without the television screen and their reasons why. All data were collected anonymously and statistical analysis was performed using the Mann–Whitney test.

## Results

**Figure 1:**
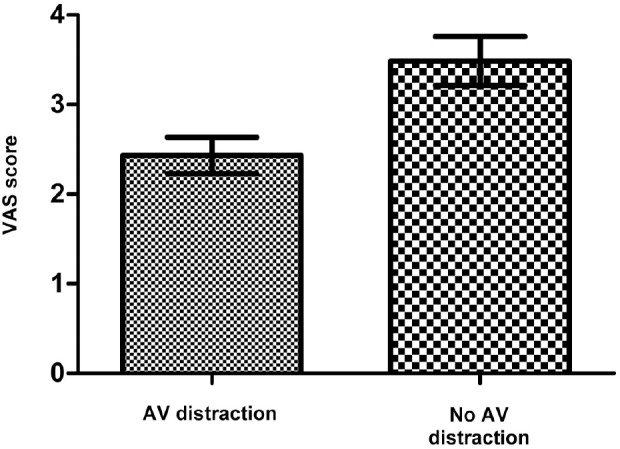
Comparison of visual analogue scale (VAS) scores for pain experienced by patients undergoing aural microsuction with and without audiovisual (AV) distraction. Error bars indicate standard deviation from mean for the two groups

A total of 37 patients were included in the study. There were 20 men and 17 women with a mean age of 66 years (range: 47–85 years). The mean pain score on the VAS for patients viewing their aural microsuction procedure was 2.432 (standard deviation [SD]: 0.203), with a range of 0–5. This compared with a mean pain score of 3.486 (SD: 0.273) for the same patients who did not view their procedure, with a range of 1–6. This difference in average pain scores was analysed using the non-parametric Mann–Whitney test and was found to be statistically significant (*p*=0.003) (Fig 1).

Patients were also asked to comment on whether they had a preference for watching their procedure on a television screen. A majority of 24 patients (65%) documented that they preferred to view their microsuction procedure. Only nine patients (24%) stated they preferred not to observe their procedure and four patients (11%) said they did not have a preference. Of those patients who preferred to view their procedure, the common reasons cited for this included that the television screen helped to distract them, made the procedure less painful and more relaxing. Some also said they found it interesting to observe what was being done and found it helpful to supplement their understanding of the procedure.

## Discussion

There have been a number of studies evaluating the use of different distraction techniques to aid pain management for a variety of minimally invasive procedures. The concept of using ‘distraction’ to reduce pain perception is thought to be related to the central cognitive processing requirement that is needed for the distraction task. McCaul and Malott postulated a ‘capacity model for attention and pain’.^9^ They proposed that pain perception requires attention to the pain source and central processing. However, attention capacity is limited and a distraction task may therefore draw away attention from the painful stimulus, thereby interfering with pain processing. The degree to which a distraction task interferes effectively with pain processing should be determined by the amount of attention resources it uses.

There is little known regarding the specific characteristics of individual distraction tasks and which will be optimal in pain management outcomes. Nevertheless, as pain processing is a central resource, it is considered that the distraction task must also engage central cognitive resources, in order to provide redirection of attention.^10^ Some studies have attempted to compare the effect of passive distraction tasks with interactive tasks to evaluate whether either type is superior in raising pain tolerance.^11,12^ Unfortunately, such comparisons are limited by numerous methodological confounds as two such different distraction tasks demand the function of multiple different attentional processes.

## Conclusions

We sought to evaluate the effect of AV distraction on patients’ pain perception during aural microsuction. Patients commonly find this routine outpatient procedure uncomfortable, distressing and painful, largely owing to the loud noise that it generates close to their ears, which are inherently a highly sensitive organ. The results of our study have shown a significant reduction in the pain experienced by patients when able to view their microsuction on a television screen, in keeping with a clear majority of patients stating that they preferred to view their procedure. Patients reported to prefer this distraction technique as it made them feel more involved, distracted them from the physical aspects of microsuction and improved their understanding of the procedure, thereby making it more acceptable. AV distraction may therefore provide a simple tool to help improve patients’ experience and acceptance of this procedure.
